# Security Architecture and Protocol for Trust Verifications Regarding the Integrity of Files Stored in Cloud Services †

**DOI:** 10.3390/s18030753

**Published:** 2018-03-02

**Authors:** Alexandre Pinheiro, Edna Dias Canedo, Rafael Timoteo de Sousa Junior, Robson de Oliveira Albuquerque, Luis Javier García Villalba, Tai-Hoon Kim

**Affiliations:** 1 Cybersecurity INCT Unit 6, Decision Technologies Laboratory—LATITUDE, Electrical Engineering Department (ENE), Technology College, University of Brasília (UnB), Brasília-DF, CEP 70910-900, Brazil; alexandre.pinheiro@redes.unb.br (A.P.); desousa@unb.br (R.T.d.S.J.); robson@redes.unb.br (R.d.O.A.); 2 Department of Computer Science, University of Brasília (UnB), P.O. Box 4466—Brasília-DF, CEP 70910-900, Brazil; ednacanedo@unb.br; 3 Group of Analysis, Security and Systems (GASS), Department of Software Engineering and Artificial Intelligence (DISIA), Faculty of Computer Science and Engineering, Office 431, Universidad Complutense de Madrid (UCM), Calle Profesor José García Santesmases, 9, Ciudad Universitaria, 28040 Madrid, Spain; 4 Department of Convergence Security, Sungshin Women’s University, 249-1 Dongseon-Dong 3-ga, Seoul 136-742, Korea; taihoonn@daum.net

**Keywords:** cloud computing, cloud data storage, proof of integrity, services monitoring, trust

## Abstract

Cloud computing is considered an interesting paradigm due to its scalability, availability and virtually unlimited storage capacity. However, it is challenging to organize a cloud storage service (CSS) that is safe from the client point-of-view and to implement this CSS in public clouds since it is not advisable to blindly consider this configuration as fully trustworthy. Ideally, owners of large amounts of data should trust their data to be in the cloud for a long period of time, without the burden of keeping copies of the original data, nor of accessing the whole content for verifications regarding data preservation. Due to these requirements, integrity, availability, privacy and trust are still challenging issues for the adoption of cloud storage services, especially when losing or leaking information can bring significant damage, be it legal or business-related. With such concerns in mind, this paper proposes an architecture for periodically monitoring both the information stored in the cloud and the service provider behavior. The architecture operates with a proposed protocol based on trust and encryption concepts to ensure cloud data integrity without compromising confidentiality and without overloading storage services. Extensive tests and simulations of the proposed architecture and protocol validate their functional behavior and performance.

## 1. Introduction

Companies, institutions and government agencies generate large amounts of digital information every day, such as documents, projects and transaction records. For legal or business reasons, this information needs to remain stored for long periods of time.

Due to the popularization of cloud computing (CC), its cost reduction and an ever-growing supply of cloud storage services (CSS), many companies are choosing these services to store their sensitive information. Cloud computing’s advantages include scalability, availability and virtually unlimited storage capacity. However, it is a challenge to build safe storage services, mainly when these services will run in public cloud infrastructures and will be managed by service providers under conditions that are not fully trustworthy.

Data owners often need to keep their stored data for a long time, though it is possible that they rarely will have to access it. Furthermore, some data could be stored in a CSS without its owner having to keep the original copy. However, in these situations, the storage service reliability must be considered, because even the best services sometimes fail [1], and since the lose of these data or their leakage can bring significant business or legal damage, the issues of integrity, availability, privacy and trust need to be answered before the adoption of the CSS.

Data integrity is defined as the accuracy and consistency of stored data. These two properties indicate that the data have not changed and have not been broken [2]. Moreover, besides data integrity, a considerable number of organizations consider both confidentiality and privacy requirements as the main obstacles to the acceptance of public cloud services [2]. Hence, to fulfill these requirements, a CSS should provide mechanisms to confirm data integrity, while still ensuring user privacy and data confidentiality.

Considering these requirements, this paper proposes an architecture for periodically monitoring both the information stored in the cloud infrastructure and the contracted storage service behavior. The architecture is based on the operation of a proposed protocol that uses a third party and applies trust and encryption means to verify both the existence and the integrity of data stored in the cloud infrastructure without compromising these data’s confidentiality. Furthermore, the protocol was designed to minimize the overload that it imposes on the cloud storage service.

To validate the proposed architecture and its supporting protocol, a corresponding prototype was developed and implemented. Then, this prototype was submitted to testing and simulations by means of which we verified its functional characteristics and its performance.

This paper is structured as follows: Section 2 reviews the concepts and definitions of cloud computing, encryption and trust; Section 3 presents works related to data integrity in the cloud; Section 4 describes the proposed architecture, while its implementation is discussed in Section 5. Section 6 is devoted to the experiments and respective results; the main differences between related works and the proposed architecture are discussed in Section 7. Section 8 closes this paper with our conclusions and outlines future works.

## 2. Background

Cloud computing (CC) is a model that allows convenient and on-demand network access to a shared set of configurable computational resources. These resources can be quickly provisioned with minimal management effort and without the service provider’s intervention [3]. Since it constitutes a flexible and reliable computing environment, CC is being gradually adopted in different business scenarios using several available supporting solutions.

Relying on different technologies (e.g., virtualization, utility computing, grid computing and service-oriented architecture) and proposing a new computational services paradigm, CC requires high-level management activities, which include: (a) selection of the service provider, (b) selection of virtualization technology, (c) virtual resources’ allocation and (d) monitoring and auditing procedures to comply with service level agreements (SLAs) [4].

A particular CC solution comprises several components such as client modules, data centers and distributed servers. These elements form the three parts of the cloud solution [4,5], each one with a specific purpose and specific role in delivering working applications based on the cloud.

The CC architecture is basically structured into two main layers namely, a lower and a higher resource layer, each one dealing with a particular aspect of making application resources available. The lower layer comprises the physical infrastructure, and it is responsible for the virtualization of storage and computational resources. The higher layer provides specific services, such as software as a service (SaaS), platform as a service (PaaS), and infrastructure as a service (IaaS). Each of these layers may have its own management and monitoring systems, independent of one another, thus improving flexibility, reuse and scalability [6,7].

Since CC provides access to a shared pool of configurable computing resources, its provisioning mode can be classified considering to the intended access methods and coverage of services’ availability, which yields different models of CC services’ deployment, ranging from private clouds, in which resources are shared within an owner organization, to public clouds, in which cloud providers possess the resources that are consumed by other organizations based on contracts, but also including hybrid cloud environments and community clouds [8].

The central concept of this paper’s proposal is the verification by the cloud service user that a particular property, in our case the integrity of files, is fulfilled by the cloud service provider, regardless of the mode of services’ provision and deployment, either in the form of private, public or hybrid clouds.

The verification of file integrity is performed by means of a protocol that uses contemporaneous computational encryption, specifically public key encryption and hashes, which together provide authentication of messages and compact integrity verification sequences that are unequivocally bound to each verified file (signed file hashes).

This proposed protocol is conceived to allow the user of cloud services to check whether the services provider is indeed acting as expected in regard to maintaining the integrity of the user files, which corresponds to the idea of the user monitoring the provider to acquire and maintain trust in the provider behavior in this circumstance.

Some specific aspects of trust, encryption and hashes that are considered as useful for this paper’s comprehension are briefly reviewed in the subsections below.

### 2.1. Trust

Trust is a common reasoning process for humans to face the world’s complexities and to think sensibly about everyday life possibilities. Trust is strongly linked to expectancies about something, which implies a degree of uncertainty and optimism. It is the choice of putting something in another’s hands, considering the other’s behavior to determine how to act in a given situation [9].

Trust can be considered as a particular level of subjective probability in which an agent believes that another agent will perform a certain action, which is subject to monitoring [10]. Furthermore, trust can be represented as an opinion so that situations involving trust and trust relationships can be modeled. Thus, positive and negative feedback on a specific entity can be accumulated and used to calculate its future behavior [11]. This opinion may result from direct experience or may come from a recommendation from another entity [12].

According to [13,14], trust, trust models and trust management have been the subject of various research works demonstrating that the conceptualization of computational trust allows a computing entity to reason with and about trust, and to make decisions regarding other entities. Indeed, since the initial works on the subject, such as [9,15], computational trust is recognized as an important aspect for decision-making in distributed and auto-organized applications, and its expression allows formalizing and clarifying trust aspects in communication protocols.

In [15], for instance, the notion of trust states the fact that if an entity A trusts an entity B in some respect, this means that A believes that B will behave in a certain way and will perform some action under certain specific circumstances. This leads to the possibility of conducting a protocol operation (action) that is evaluated by the entity A on the basis of what A knows about the entity B and the circumstances of the operation. This accurately corresponds to the protocol relationship established between a CC service consumer and a CC service provider, which is the focus of the present paper.

Thus, in our proposal, trust is used in the context of a cloud computing service as a means to verify specific actions performed by the participating entities in this context. Using the definitions by [15,16], we can state that in a CC service, one entity, the CC service consumer, may trust another one, the CC service provider, for actions such as providing identification to the other entity, not interfering in the other entity sessions, neither passively by reading secret messages, nor actively by impersonating other parties. Furthermore, the CC service provider will grant access to resources or services, as well as make decisions on behalf of the other entity, with respect to a resource or service that this entity owns or controls.

In these trust verifications, it is required to ensure some properties such as the secrecy and integrity of stored files, authentication of message sources and the freshness of the presented proofs, avoiding proof replays. It is required as well to present reduced overhead in cloud computing protocol operations and services. In our proposal, these requirements are fulfilled with modern robust public key encryption involving hashes, as discussed hereafter, considering that these means are adequately and easily deployed in current CC service providers and consumers.

### 2.2. Encryption

Encryption is indeed a process of converting (or ciphering) a plaintext message into a ciphertext that can be deciphered back to the original message. An encryption algorithm, along with one or more keys, is used either in the encryption or the decryption operation.

The number, type and length of the keys used depend on the encryption algorithm, the choice of which is a consequence of the security level needed. In conventional symmetric encryption, a single key is used, and with this key, the sender can encrypt a message and a recipient can decrypt the ciphered message, but the key security becomes an issue since at least two copies of the key exist, one at the sender and another at the recipient.

Oppositely, in asymmetric encryption, the encryption key and the decryption key are correlated, but different, one being a public key of the recipient that can be used by the sender to encrypt the message, while the other related key is a recipient private key allowing the recipient to decrypt the message [17]. The private key can be used by its owner to send messages that are considered signed by the owner since every entity can use the corresponding public key to verify if a message comes from the owner of the private key.

These properties of asymmetric encryption are useful for the trust verifications that in our proposal are designed for checking the integrity of files stored in cloud services. Indeed, our proposal uses encryption of hashes as the principal means to fulfill the trust requirements in these operations.

### 2.3. Hashes

A hash value, hash code or simply hash is the result of applying a mathematical one-way function that takes a string of any size as the data source and returns a relatively small and fixed-length string. A modification of any bit in the source string dramatically alters the resulting hash code after executing the hash function [18]. These one-way functions are designed to make it very difficult to deduce from a hash value the source string that was used to calculate this hash. Furthermore, it is required that it should be extremely difficult to find two source strings whose hash codes are the same, i.e., a hash collision.

Over the years, many cryptographic algorithms have been developed for hashes, for which the Message-Digest algorithm 5 (MD5) and Secure Hash Algorithm (SHA) family of algorithms can be highlighted, due to the wide use of these algorithms in the most diverse information security software packages. MD5 is a very fast cryptographic algorithm that receives as input a random-sized message and produces as output a fixed length hash with 128 bits [19].

The SHA family is composed of algorithms named as SHA-1, SHA-256, SHA-512, which differ regarding the respective security level and the output hash length, that can vary from 160 to 512 bits. The SHA-3 algorithm was chosen by the National Institute of Standards and Technology (NIST) in an international competition that aimed to replace all of the SHA family of algorithms [20].

The Blake2 algorithm is an improved version of the hash cryptographic algorithm called “Blake”, a finalist of the SHA-3 selection competition that is optimized for software applications. Blake2 can generate hash values from eight to 512 bits. The main Blake2 characteristics are: the memory consumption reduction by 32% compared to other SHA algorithms, the processing speed being greater than that of MD5 on 64-bit platforms, direct parallelism support without overhead and faster hash generation on multicore processors [21]. In our proposed validation prototype, the Blake2 algorithm was considered as a good choice due to its combined characteristics of speed, security and simplicity.

## 3. Related Work

This section presents a brief review of papers regarding the themes of computational trust applications, privacy guarantees, data integrity verification, services management and monitoring, all of them applicable to cloud computing environments.

### 3.1. Computational Trust Applications

Depending on the used approach, trust can either be directly measured by one entity based on its own experiences or can be evaluated through the use of third-party opinions and recommendations.

In [22], a trust model called “GenTrust” was proposed for peer-to-peer (P2P) systems, in which genetic algorithms are used to recognize several types of attacks and to help a well-behaved node find other trusted nodes. GenTrust uses extracted features (number of interactions, number of successful interactions, the average size of downloaded files, the average time between two interactions, etc.) that result from a node’s own interactions. However, when there is not enough information for a node to consider, recommendations from other nodes are used. Then, the genetic algorithm selects which characteristics, when evaluated together and in a given context, present the best result to identify the most trustful nodes.

Another approach is presented in [23], proposing a trust model named “Turnaround_Trust” aimed at helping clients to find cloud services that can serve them based on service quality requirements. The Turnaround_Trust model considers service quality criteria such as cost, response time, bandwidth and processor speed, to select the most trustful service among those available in the cloud.

Our approach in this paper differs from these related works since we use trust metrics that are directly related to the stored files in CC and that are paired to the cryptographic proof of these files integrity.

In [24], the proposed trust model is based on concepts such as direct trust, trust recommendation, indirect trust, situational trust and reputation to allow a node selection for trustful file exchange in a private cloud. For the sake of trust calculation, the processing capacity of a node, its storage capacity and operating system, as well as the link capacity are adopted as trust metrics that compose a set representative of the node availability. Concerning reputation, the calculation considers the satisfactory and unsatisfactory experiences with the referred node informed by other nodes. The proposed model calculates trust and reputation scores for a node based on previously-collected information, i.e., either information requested from other nodes in the network or information that is directly collected from interactions with the node being evaluated.

In the present paper, our approach is applied to both private and public CC services, with the development of the necessary architecture and secure protocol for trust verification regarding the integrity of files in CC services.

### 3.2. Integrity Verification and Privacy Guarantee

In their effort to guarantee the integrity of data stored in cloud services, many research works present proposals in the domain analyzed in this paper.

A protocol is proposed in [25] to enable a cloud storage service to prove that a file subjected to verification is not corrupted. To that end, a formal and secure definition of proof of retrievability is presented, and the paper introduces the use of sentinels, which are special blocks hidden in the original file prior to encryption to be afterward used to challenge the cloud service. In [26], based on [25], another scheme is presented where one does not need to encrypt all the data, but only a few bits per data block.

The work in [27] proposes a bipartite solution to improve privacy and integrity. The first part, called “anonymization”, initially recognizes fields in records that could identify their owners and then uses techniques such as generalization, suppression, obfuscation and the addition of anonymous records to enhance data privacy. The second part, called “integrity checking”, uses public and private key encryption techniques to generate a tag for each record on a table. Both parts are executed with the help of a trusted third party called the “enclave” that saves all generated data that will be used by the de-anonymization and integrity verification processes.

An encryption-based integrity verification method is proposed by [28]. The proposed method uses a new hash algorithm, the dynamic user policy-based hash algorithm, to calculate hashes of data for each authorized cloud user. For data encryption, an improved attribute-based encryption algorithm is used. The encrypted data and corresponding hash value are saved separately in cloud storage. Data integrity can be verified only by an authorized user and requires the retrieval of all the encrypted data and corresponding hash.

Another proposal to simultaneously achieve data integrity verification and privacy-preserving is found in [29], which proposes the use of two encryption algorithms for every data upload or download transaction. The Advanced Encryption Standard (AES) algorithm is used to encrypt client data, which will be saved in a CSS, and an RSA-based partial homomorphic encryption technique is used to encrypt AES encryption keys that will be saved in a third party entity together with a hash of the file. Data integrity is verified only when a client downloads one file.

In [30], a data integrity auditing protocol is proposed to allow the fast identification of corrupted data using homomorphic cipher-text verification and a recoverable coding methodology. Checking the integrity of outsourced data is done periodically by either a trusted or untrusted entity. The adopted methodology aims at reducing the total auditing time and the communication cost.

The work of [31] presents a security model for public verification and assurance of stored file correctness that supports dynamic data operation. The model guarantees that no challenged file blocks should be retrieved by the verifier during the verification process and no state information should be stored at the verifier side between audits. A Merkle hash tree (MHT) is used to save the authentic data value hashes, and both the values and positions of data blocks are authenticated by the verifier.

Our proposal in this paper differs from these described proposals since we introduce the idea of trust resulting from file integrity verifications as an aggregate concept to evaluate the long-term behavior of a CSS and including most of the requirements specified in these other proposals, such as hashes of file blocks, freshness of verifications and integrated support for auditing by an independent party. Further discussion on theses differences is presented in Section 7 based on the results coming from the validation of our proposal.

### 3.3. Management and Monitoring of CSS

Some other research works were reviewed since their purpose is to provide management tools to ensure better use of the services offered by CSS providers, as well as monitoring functions regarding the quality of these services, thus allowing one to generate a ranking of these providers.

An approach to autonomous data management within CSS is presented in [32]. This approach proposes a high-level service that helps users to better manage data distributed in multiple CSS. The proposed solution is composed of a framework that consists of three components named MeasureTool, DistributeTool and CollectTool. Each component is respectively responsible for performing monitoring processes for measuring the performance, splitting and distributing file chunks between different CSS and retrieving split parts of a required file. Both historical performance and latest performance values are used for CSS selection and to define the number of file chunks that will be stored in each CSS.

Furthermore, in [32] the use of cloud infrastructure services is proposed to execute applications on mobile data stored in CSS. In this proposal, the services for data management are run in one or more IaaS systems that keep track of the user storage area in CSS and execute the data manipulation processes when new files appear. The service running on an IaaS cloud downloads the user data files from the CSS, executes the necessary application on these files and uploads the modified data to the CSS. This approach permits overcoming the computing capacity limitations of mobile devices.

The quality of services (QoS) provided by some commercial CSS is analyzed in [33]. For this, a measurement study is presented where important aspects such as transfer speed (upload/download), behavior according to client geographic location, failure rate and service variability related to file size, time and account load are broadly explored. To perform the measurement, two platforms are employed, one with homogeneous and dedicated machines and the other with shared and heterogeneous machines distributed in different geographic locations. Furthermore, the measurement is executed using its own CSS REST interfaces, regarding mainly the methods PUT and GET, respectively used to upload and download files. The applied measurement methodology is demonstrated to be efficient and permits one to learn important characteristics about the analyzed CSS.

Our contributions in this paper comprise the periodic monitoring of files stored in the cloud, performed by an integrity checking service that is defined as an abstract role so that it can operate independently either the CSS provider or its consumer, preserving the privacy of stored file contents, and operating according to a new verification protocol. Both the tripartite architecture and the proposed protocol are described hereafter in this paper.

## 4. Proposed Architecture and Protocol

This section presents the proposed architecture that defines roles that work together to enable periodic monitoring of files stored in the cloud. Furthermore, the companion protocol that regulates how these roles interact with one another is detailed and discussed.

The architecture is composed of three roles: (i) Client; (ii) Cloud Storage Service (CSS) and (iii) Integrity Check Service (ICS).The Client represents the owner of files that will be stored by the cloud provider and is responsible for generating the needed information that is stored specifically for the purpose of file integrity monitoring.

The CSS role represents the entity responsible for receiving and storing the client’s files, as well as receiving and responding to challenges regarding file integrity that come from the ICS role.

The ICS interfaces either with the Client of the CSS, so it acts as the responsible role for information regarding the Client files that are stored by the CSS and uses this information to constantly monitor the Client files’ integrity by submitting challenges to the CSS and later validating the responses of the CSS to each verification challenge.

### 4.1. The Proposed Protocol

The trust-oriented protocol for continuous monitoring of stored files in the cloud (TOPMCloud) was initially proposed in [34,35]. Then, it was further developed and tested giving way to the results presented in this paper.

The TOPMCloud objective is to make the utilization of an outsourced service possible to allow clients to constantly monitor the integrity of their stored files in CSS, without having to keep original file copies or revealing the contents of these files.

From another point of view, the primary requirement for the proposed TOPMCloud is to prevent the CSS provider from offering to and charging a client for a storage service that in practice is not being provided. Complementary requirements comprise low bandwidth consumption, minimal CSS overloading, rapid identification of a misbehaving service, strong defenses against fraud, stored data confidentiality and utmost predictability for the ICS.

**Figure 1:**
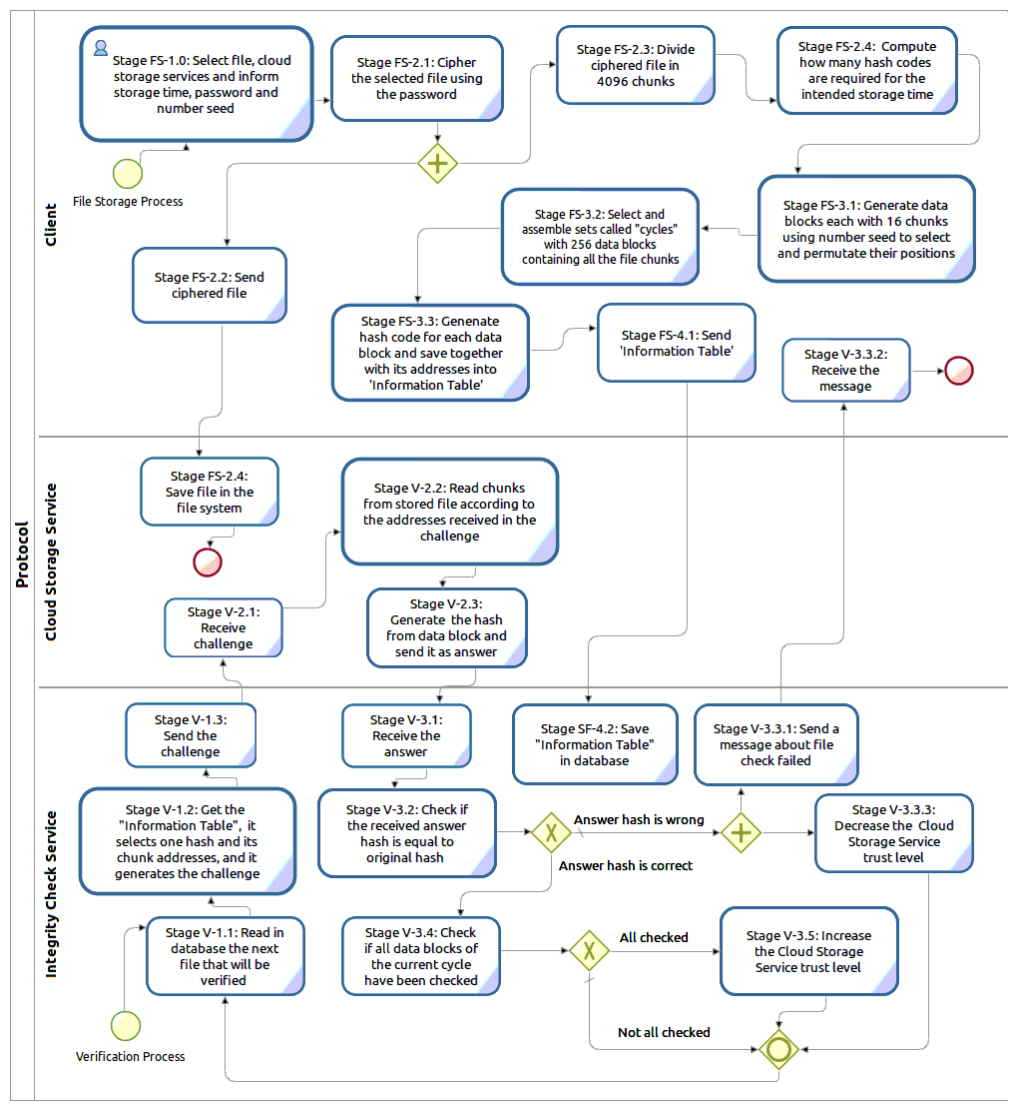
Trust-oriented protocol for continuous monitoring of stored files in the cloud (TOPMCloud) processes.

To respond to the specified requirements, TOPMCloud is designed with two distinct and correlated execution processes that are shown together in Figure 1. The first one is called “File Storage Process” and runs on demand from the Client that is this process starting entity. The second is the “Verification Process”, which is instantiated by an ICS and is continuously executed to verify a CSS. An ICS can simultaneously verify more than one CSS by means of parallel instances of the Verification Process.

The File Storage Process starts in the Client with the encryption of the file to be stored in the CSS. This first step, which is performed under the control of the file owner, is followed by the division of the encrypted file into 4096 chunks. These chunks are randomly permuted and are selected to be grouped into data blocks, each one with 16 distinct file chunks, and the position or address of each chunk is memorized. Then, hashes are generated from these data blocks. Each hash together with the set of its respective chunk addresses are used to build a data structure named the Information Table, which is sent to the ICS.

The selection and distribution of chunks used to assemble the data blocks are done in cycles. The number of cycles will vary according to the file storage period. Each cycle generates 256 data blocks without repeating chunks. The data blocks generated in each cycle contain all of the chunks of the encrypted file (256 * 16 = 4096).

The chosen values, 4096, 16 and 256, come from a compromise involving the analysis of the protocol in the next subsections and the experimental evaluation that is presented in Section 6 of this paper. Therefore, these values represent choices that were made considering the freshness of the information regarding the trust credited to a CSS, the time for the whole architecture to react to file storage faults, the required number of verifications to hold the trust in a CSS for a certain period of time, as well as the expected performance and the optimization of computational resources and network capacity consumption. The chosen values are indeed parameters in our prototype code, so they can evolve if the protocol requirements change.

The Verification Process in the ICS starts with the computation of how many files should be verified and how many challenges should be sent to a CSS, both numbers being calculated according to the trust level assigned to the CSS. Each stored hash and its corresponding chunk addresses will be used only once by the ICS to send an integrity verification challenge to the CSS provider.

In the CSS, the stored file will be used to respond to the challenges coming from the ICS. On receiving a challenge with a set of chunk addresses, the CSS reads the chunks from the stored file, assembles the data block, generates a hash from this data block and sends this hash as the challenge answer to the ICS.

To finalize the verification by the ICS, the hash coming in the challenge answer is compared to the original file hash, and the result activates the trust level classification process. For this process, if the compared hashes are equal, this means that the verified content chunks are intact in the stored file in the CSS.

### 4.2. Trust Level Classification Process

The trust level is evaluated as a real value in the range (−1, +1), with values from −1, meaning the most untrustful, to +1, meaning the most trustful, thus constituting the classification level that is attributed by the ICS to the CSS provider.

In the ICS, whenever a file hash verification process fails, the trust level of the verified CSS is downgraded, according to the following rules: when the current trust level value is greater than zero, it is set to zero (the ICS reacts quickly to a misbehavior from a CSS that was considered up to the moment as trustful); when the trust value is in the range between zero and −0.5, it is reduced by 15%; otherwise, the ICS calculates the value of 2.5% from the difference between the current trust level value and −1, and the result is subtracted from trust level value (the ICS continuously downgrades a CSS that is still considered untrustful). These calculations are shown in Algorithm 1.

**Algorithm 1:** Pseudocode for computing the TrustLevel in the case of hash verification failures.
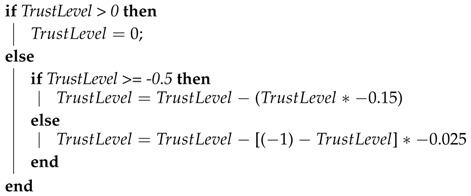


Conversely, whenever a checking cycle is completed without failures (all data blocks of a file have been checked without errors), the trust level assigned to a CSS is raised. If the current trust level value is less than 0.5, then the trust level value is raised by 2.5%. Otherwise, the ICS calculates the value of 0.5% from the difference between one and the current trust level value, and the result is added to the trust level value. These calculations are shown in Algorithm 2. This means that initially, we softly redeem an untrustful CSS, while we exponentially upgrade a redeemed CSS and a still trustful CSS. 

**Algorithm 2:** Pseudocode for computing the TrustLevel in the case of hash verification successes.
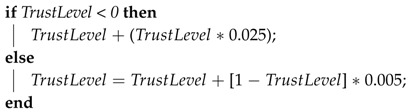


Again, these chosen thresholds and downgrading/upgrading values come from the experimental evaluation that is presented in Section 6, based on performance and applicability criteria. They are indeed parameters in our prototype code, so they can evolve if the protocol requirements change.

### 4.3. Freshness of the Trust Verification Process

Since it is important to update the perception that a Client has about a CCS provider, the observed values of trust regarding a CSS are also used to determine the rhythm or intensity of verifications to be performed for this CSS.

Thus, the freshness of results from the trust verification process is assured by updating in the ICS the minimum percentage values of the number of stored files to be verified in a CSS, as well as the minimum percentages of data blocks that should be checked. We choose to present these updates by day, though again, this is a parameter in our implemented prototype.

Consequently, according to the observed trust level for a CSS, the number of files and the percentage of these file contents checked in this CSS are set as specified in Table 1. In this table, the extreme values one and −1 should respectively represent blind trust and complete distrust, but they are not considered as valid for our classification purposes, since we expect trust to be an ever-changing variable, including the idea of redemption.

Whenever the trust value equals zero, as a means to have a decidable system, a fixed value must be artificially assigned to it to preserve the dynamics of evaluations. Thus, if the last verified result is a positive assessment, the value +0.1 is assigned to the observed trust; otherwise, if a verification fault has been observed, the assigned value is −0.1.

### 4.4. Variation of the Trust Level Assigned to the Cloud Storage Service

According to the TOPMCloud definition, the trust level assigned to a CSS always grows when a file-checking cycle is finished without the ICS detecting any verification failures during this cycle. Considering this rule, the first simulations regarding the evolution of trust in the ICS were used to determine the maximum number of days needed for the ICS to finish a checking cycle for a file stored in a CSS. The conclusion of a checking cycle indicates that each of the 4096 file chunks was validated as a part of one of the data blocks that are checked by means of the 256 challenges submitted by the ICS to the CSS.

The projected time for our algorithm to finish a file-checking cycle can vary between a minimum and a maximum value depending on the number of files simultaneously monitored by the ICS on a CSS. However, the checked file size should not significantly influence this time because the daily number of checked data blocks on a file is a percentage of the file size, as defined in Table 1.

**Figure 2:**
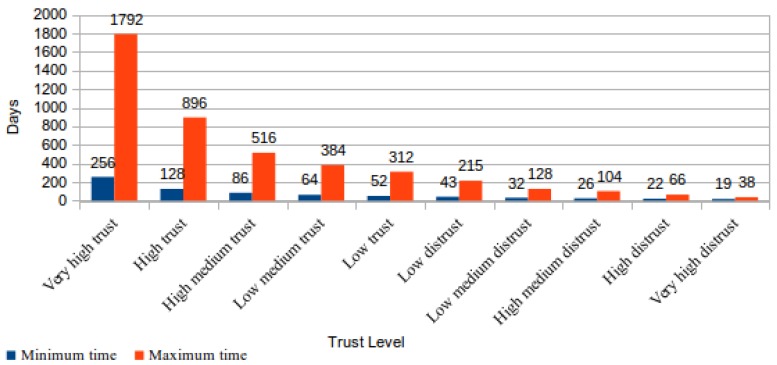
Time required to complete a file-checking cycle.

By means of mathematical calculations, it is possible to determine that in a CSS classified with a “very high distrust” level, i.e, the worst trust level, the maximum time to finish a checking cycle is 38 days. Comparatively, in a CSS classified with a “very high trust” level, i.e., the best trust level, the time to finish a checking cycle can reach 1792 days. Figure 2 shows the maximum and the minimum number of days required to finish a file-checking cycle for each trust level proposed in TOPMCloud.

Notwithstanding the mathematical calculations regarding the proposed protocol’s maximum time required to finish a file-checking cycle, it is noticeable that this time can increase if the ICS or the CSS servers do not have enough computational capacity to respectively generate or to answer the necessary protocol challenges for each day. Furthermore, the file-checking cycle depends on the available network bandwidth and can worsen if the network does not support the generated packet traffic. This situation can occur when the number of CSS stored files is very large.

The variation of the time to conclude the checking cycle, according to the trust level assigned to the CSS, comes from the different number of data blocks verified per day. This variation aims to reward cloud storage services that historically have no faults, thus minimizing the consumption of resources such as processing capacity and network bandwidth. Moreover, this feature allows our proposed architecture to prioritize the checking of files that are stored in CSS providers, which have already presented faults. Consequently, this feature reduces the requested time to determine if other files were lost or corrupted.

**Figure 3:**
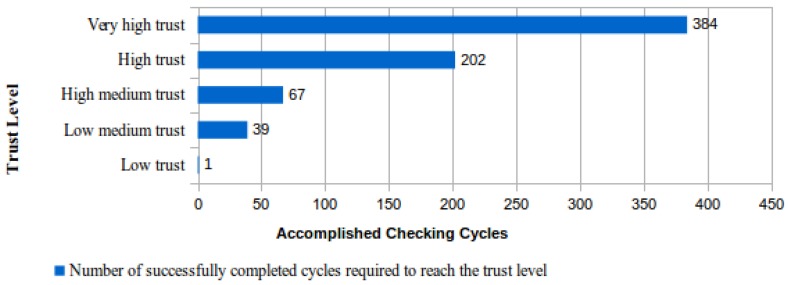
Expected best performing trust level evolution for a CSS.

Another interesting characteristic of the proposed protocol was analyzed with calculations that were realized to determine the number of file cycles concluded without identifying any fault so that the trust level assigned to a CSS raises to the highest trust level foreseen in Table 1, the “very high trust”. Figure 3 presents the results of this analysis using as a starting point the “not evaluated” situation, which corresponds to a trust level equal to zero assigned to a CSS.

From the analysis of the results shown in Figure 2 and Figure 3, it could be concluded that the requested time for a CSS to obtain the maximum trust level is so large that it will be practically impossible to reach this level. This conclusion is easily obtained using the maximum number of days needed to finish a checking cycle for the “high trust” level (896) multiplied by the number of successfully concluded cycles to reach the level of “very high trust” (384 −202 = 182). The result of this calculation is 163.072 days (182 * 896), which is approximately 453 years.

Although this is mathematically correct, in practice, this situation would never occur. The simple explanation for this fact is related to the number of files that have been simultaneously monitored by the ICS in the CSS. The maximum expected time for the file-checking cycle conclusion only occurs when the number of monitored files in a CSS, classified with the level “high trust”, is equal to 25 or a multiple of this value. According to Table 1, this is due to the fact that, at the “high trust” level, it is required that 16% of the file content should be checked by day. The maximum time spent in file checking only occurs when the result of this file percentage calculation is equal to an integer value. Otherwise, the result is rounded up, thus increasing the percentage of files effectively checked.

Indeed, if the ICS is monitoring exactly 25 files in a CSS that is classified with the “high trust” level and supposing that these files were submitted to CSS in the same day, the checking cycles for this set of files will finish in 896 days. Since in a period of 896 days, there are 25 concluded cycles, then about 20 years are needed for the CSS to attain the 182 cycles requested for reaching the next level, the “very high trust”. However, this situation worsens if the number of considered files decreases. For instance, considering the “high trust” level, if there are only six files being monitored, then the time to attain the next level exceeds 65 years.

**Figure 4:**
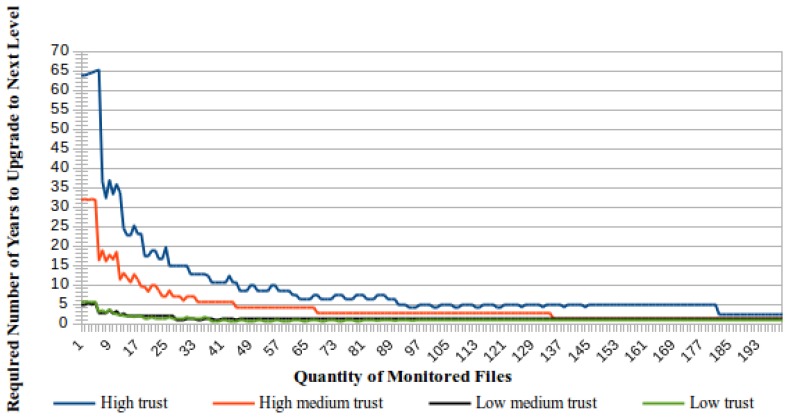
Time to upgrade the trust level according to the number of monitored files.

Figure 4 presents a comparative view of the time required to upgrade to the next trust level according to the number of monitored files. In general, less time will be required to increase the trust level if there are more monitored files.

As can be seen in Figure 4, the best case is obtained when the number of monitored files is equal to the required number of successfully concluded cycles to upgrade to the next trust level. For this number of files, the time required to increase the trust level is always equal to the time needed to conclude one checking cycle.

Opposite to the trust level raising curve that reflects a slow and gradual process, the trust level reduction is designed as a very fast process. The trust value assigned to the CSS always decreases when a challenge result indicates a fault in a checked file.

**Figure 5:**
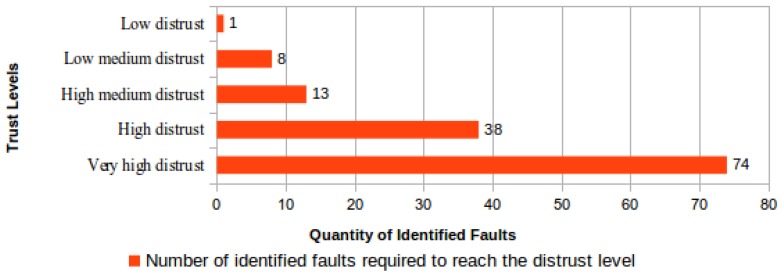
Number of file-checking failures needed to downgrade to each distrust level.

To evaluate the proposed process for downgrading the measured trust level, calculations were performed aiming to determine how many file-checking failures are needed for a CSS to reach the maximum distrust level. Any trust level between “very high trust” and “low trust” could be used as the starting point to these calculations. Then, when a challenge-response failure is identified, the trust value is changed to zero and the CSS is immediately reclassified to the “low distrust” level. From this level to the “very high distrust” level, the number of file-checking failures required to reach each next distrust level is shown in Figure 5.

**Figure 6:**
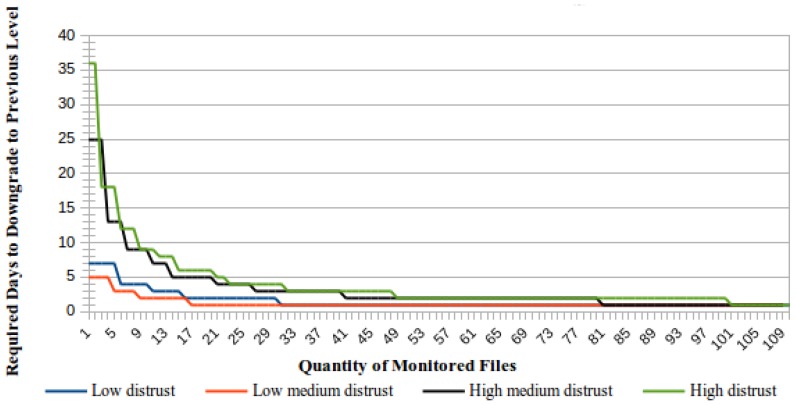
Trust level downgrade according to the number of monitored files.

Similarly to the trust level raising process, the required minimum time to downgrade to a distrust level is determined by the number of simultaneously-monitored files. Figure 6 presents a comparative view of the required minimum time to downgrade a CSS considering that all monitored files are corrupted and that failures will be identified upon the ICS receiving the first unsuccessful challenge response from the CSS.

An important difference between the process to downgrade the trust level assigned to a CSS and the opposite process to upgrade this trust level is that the downgrade time is preferably presented as a number of days, whereas the upgrade time is preferably presented in years. As shown in Figure 6, the minimum time to downgrade a trust level will be one day when the number of monitored files is equal to or greater than the number of identified challenge failures required to downgrade a trust level according to Figure 5.

## 5. Architecture Implementation

The implementation of the architecture was organized as a validation project comprising three phases. the first phase was devoted to the processes under the responsibility of the client in our proposal. The second and the third phases were respectively aimed at implementing the processes under the ICS responsibility and the processes under the responsibility of the CSS. Hence, a completely functional prototype was used for the validation of the proposed architecture and protocol.

In each implementation phase, one application was developed using Java Enterprise Edition (Java EE) components, such as Java Persistence API (JPA), Enterprise JavaBeans (EJB), Contexts and Dependency Injection (CDI) and Java API for XML Web Services (JAX-WS) [36]. A desktop application was developed for the Client, while two web service applications were developed respectively for the ICS and CSS. The chosen application server was Glassfish 4 [37], and the chosen database management system (DBMS) was PostgreSQL [38].

These platforms were chosen to take into consideration the distributed characteristics of the proposed architecture and the need for continuous and asynchronous protocol communications between the predicted roles in this architecture. Thus, the choice of Java EEwas determined by its usability for the implementation of web services, task scheduling, event monitoring and asynchronous calls. Both the Glassfish application server and the PostgreSQL DBMS were chosen because they are open source applications and fully meet the developed application needs.

### 5.1. Client Application

The Client application’s main tasks are: to encrypt a file and to send it to one or more CSS, to split and select the file chunks, to assemble the data blocks and to generate their hashes, to group them in cycles, to generate the Information Table and to send this table to the ICS. In our prototype, the client application also allows one to control the inventory of stored files in each CSS, to store the cryptographic keys, to look for pieces of information about the verification process and the file integrity in each CSS, to retrieve a file from a CSS, confirming its integrity and deciphering its contents.

**Figure 7:**
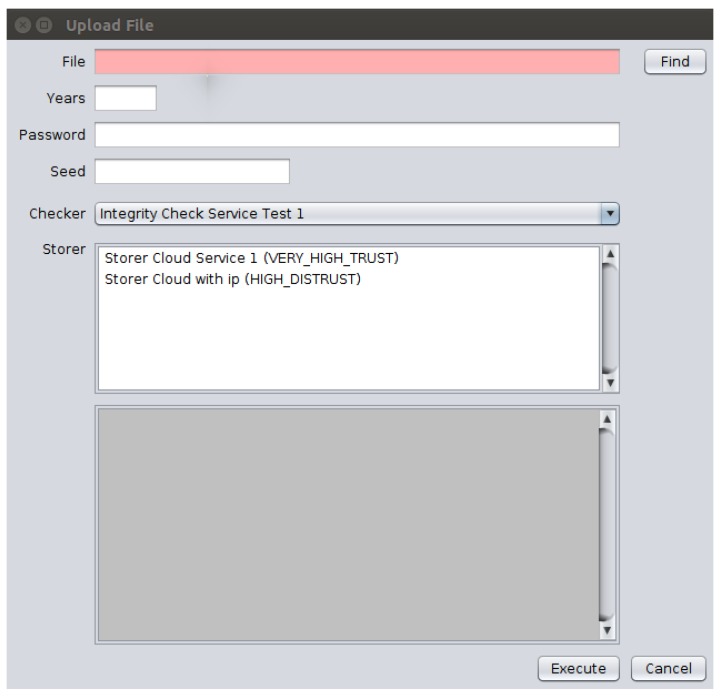
The client interface showing the “Upload File” function.

These functions are accessible in the Client application by means of a graphical interface through which the user selects files from the file system and at least one CSS to store the chosen files, as well as an ICS to verify the storage service used for these files. This same interface allows the client to inform about both the password to be used in the file encryption process and the number of years corresponding to the period to keep the file stored in the CSS. Furthermore, a numerical seed is given to add entropy to the process of choosing the chunks that will compose each data block. Figure 7 shows the application interface with the implemented “Upload File” function.

In this prototype, the list of available CSS comes from previous registration activity, by means of which the user first registers the ICS with which it maintains a service level agreement. After an ICS has been selected, the Client application obtains a list of CSS from the selected ICS web service. This initial process requires the client user to maintain some contractual relationship regarding file storage verifications involving the Client application, the registered ICS and the selected CSS. Then, the Client user is informed about the current trust level assigned by the ICS to each concerned CSS.

The file encryption process is performed using resources available in the “javax.crypto” package [39], using the AES cryptographic algorithm [40], a 256-bit key and the cipher-block chaining (CBC) operation [41].

The process of sending an encrypted file to a CSS was implemented using Java threads so that it is possible to simultaneously initiate the uploading of the encrypted file to each selected CSS. Furthermore, by means of threads, it is possible to proceed with the next steps in parallel, without the need to wait for a complete file upload, an operation that takes a variable duration according to the file size and the network characteristics.

The calculations of either the number of cycles and the chunk distribution is executed according to the number of years that the file must be stored by the CSS and monitored by the ICS. Although the number of used cycles should vary according to the trust level assigned to the CSS, in our validation prototype for these calculations, we choose to consider the worst case value, corresponding to the “high distrust” level.

Each chunk address code is obtained through the SHA1PRNG algorithm, a pseudo-random number generator, executed by “SecureRandom”, a class from the “java.security” package [39]. To produce the hashes, the cryptographic function Blake2 [21] was chosen due to its speed, security and simplicity.

**Figure 8:**
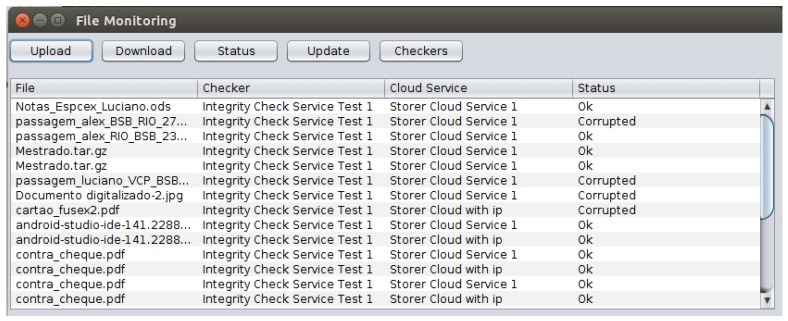
The monitoring module entry screen.

Also in our prototype, the monitoring module shown in Figure 8 was developed to provide a practical tool for the user to access functions such as: to manage the inventory of stored files in a set of CSS providers, to check the file status assigned by the ICS according to results from verifications of the CSS, to download files from the CSS and to decipher the downloaded files.

**Figure 9:**
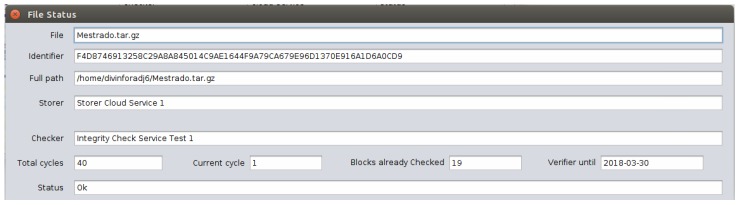
File status query screen.

In the monitoring module, the “Status” button shows information such as: the file source folder, the identification hash, the name of the CSS where the file is stored, the name of the ICS responsible for monitoring this CSS, the number of concluded checking cycles, the number of cycles that is currently being performed, the number of data blocks already checked in the current cycle, the ICS monitoring period for the last day and its current status. Figure 9 shows the file status query screen.

### 5.2. Integrity Checking Service Application

The ICS implementation comprises a web service application called “VerifierService” that presents the following functionalities: to receive the File Information Table submitted by the Client application, to select and send challenges regarding monitored files to the CSS that host them, to manage pending responses to challenges, to receive challenge responses from the CSS, to update the CSS trust levels, to receive and to answer to requests for the monitored file status, to receive and to answer requests for information about the CSS with which there is a monitoring agreement.

In the File Information Table received from the Client application, the CSS is represented by the identifier of the monitoring contract between the ICS and the CSS. For each CSS contract in the information table, the ICS application saves a new record in a table named “archives”, so that the monitoring of one file copy does not interfere with the monitoring of other copies. The following information is stored in the archives table: a file identifier hash, the chunk size in bytes, the number of generated cycles and the contract identifier that defines the CSS where the file is stored.

For each data block hash received by means of the information table coming from a Client, a new record is generated by the ICS application in a table named “blocks”. The following information is stored in this table: the data block hash, the chunk address codes that compose this block, the cycle number to which it belongs and the "archives" record identifier.

The process of selecting, generating and sending challenges is an activity performed periodically (in our prototype, daily) by the ICS. This process comprises the following actions: selecting files to be checked in each CSS, selecting data blocks to be checked in each file, generating challenges and sending the challenges to the CSS.

The trust level assigned to a CSS will be decremented whenever the ICS identifies a corrupted data block in a file. Conversely, the trust level is incremented after all data blocks from the same cycle have been the object of challenges to the CSS, and all the answers to these challenges confirm the integrity of the checked blocks. Other results obtained in a file marked as corrupted, whether positive or negative, will simply be ignored.

### 5.3. Cloud Storage Service Application

The CSS application was developed as a web service named “StorerWebService”. This application provides a service capable of receiving challenges from an ICS, processing them and sending back to the ICS the challenge responses.

The CSS application in our prototype implementation includes the following functionalities: to receive challenges from an ICS and storing them in the CSS database, to monitor the database and process the pending challenges, to store the responses in the database and to monitor the database to find and send pending responses to the ICS. Furthermore, the application also includes features for uploading and downloading files to simulate the features normally available in a CSS.

The challenge-receiving functionality is provided in ICS by means of a method called “asyncFileCheck” that gets as the input parameter an object of the class called “ChallengeBean”. This object contains the attributes “identifier”, “addressCodes”, “chunkLength”, “responseUrl” and “id”, which respectively represent: the file identifier hash, the array with the set of chunk addresses codes to be read in the file and that will compose the data block on which the response hash will be generated, the chunk size in bytes, the ICS web service URL responsible for receiving the challenge answers and the challenge identifier.

After receiving a challenge, the information is extracted from the ChallengeBean object and is stored in the CSS database, where it gets the “Waiting for Processing” status. The advantage of storing the received challenges for further processing is related to the organization of our general architecture for saving computational resources. This CSS asynchronous procedure prevents the ICS from needing to keep a process locked awaiting a response from the CSS for each submitted challenge, considering that the required time to process a challenge varies according to the checked file size, the number of simultaneously-received challenges and the CSS computational capacity.

Another designed consequence of this model is the possibility of performing load distribution since the services responsible for receiving, processing and responding to the challenges can be provided by totally different hardware and software infrastructures. The only requirement is that all infrastructure components must share access to the same database.

The response hash is generated from a data block assembled with file chunks that are read from the file being checked. The used chunks are those referenced by the 16 chunk addresses defined in the “addressCodes” attribute of the “challenge” object. These address codes are integers ranging from zero to 4095 that are multiplied by the chunk size to obtain the address of the first byte of each chunk in the file.

After the completion of the chunk reading task, the obtained data are concatenated, forming a data block. From this data block, a 256-bit hash is generated using the Blake2 hash cryptographic function [21]. The generated hash is saved in the database while waiting to be sent back to the ICS. A specific process monitors the pendent hash responses and sends it as challenge answers to ICS.

## 6. Experimental Validation

This section describes the setup used to perform the experiments designed to evaluate the performance, efficiency and efficacy of the proposed protocol TOPMCloud. Then, the results of the experimental validation are presented and discussed.

### 6.1. Experimental Setup

Our experimental environment comprises five similar virtual machines (VM), each one running with 12 GB memory, 200 GB of hard disk space, running under the operating system Ubuntu Server 14.04 LTS Server. All of them were set to run in a private cloud hosted by the Decision Technologies Laboratory at the University of Brasília, Brazil. Since this setup presents common functionalities that are found in most commercial CSS providers, it is considered as a configuration that adequately represents the utilization of these services as provided by commercial cloud services. The basic operating system functions that are required from the CSS provider are file access operations and hash calculations, which are commonly available in cloud computing services and object class libraries. Otherwise, these functions can be easily deployed by commands from the cloud services client. These VMs are configured to perform the three roles designed in our architecture, with the following distribution of services in the VM set: one VM holds the Client role; one VM performs the Integrity Check Service (ICS) role; and the remaining three VMs hold the Cloud Storage Services (CSS) role.

The experiments were realized using four files with different sizes (2.5, 5, 10 and 15 GB). For each file, an information table was generated considering the utilization of the file storage service and its monitoring during five different time periods (1, 5, 10, 20 and 30 years). Files with diverse content types and formats, such as International Organization for Standardization (ISO) 9660, XenServer Virtual Appliance (XVA) and Matroska Video (MKV) , were used. For each considered time period, cryptographic keys with the same size were used, but with different and aleatory values, so that each generated encrypted file was completely distinct from other files generated from the same origin file.

With the described configuration operating during three months, logs were generated from the ICS monitoring process verifying files stored in the three CSS providers. In total, sixty files were monitored by the ICS, twenty of them stored in each CSS. In this period, some CSS servers were randomly chosen to be turned off and after a while to be switched on again, in order to simulate fault conditions.

In order to evaluate the behavior of the proposed protocol, some experiments were realized with contextual modifications, including the deliberate change of file contents and the change of trust levels assigned to each CSS.

### 6.2. Submission of Files

Our experiments begin by observing the performance during the process of preparation and submission of files to CSS and the related transmission of their respective information tables to the ICS. The steps of this process have their duration time measured so that we collect observations regarding the following tasks: encrypting the source file to an encrypted file, hashing this encrypted file, computing cycles and distributing chunks on data blocks, hashing these data blocks and finally sending the information table to ICS.

**Figure 10:**
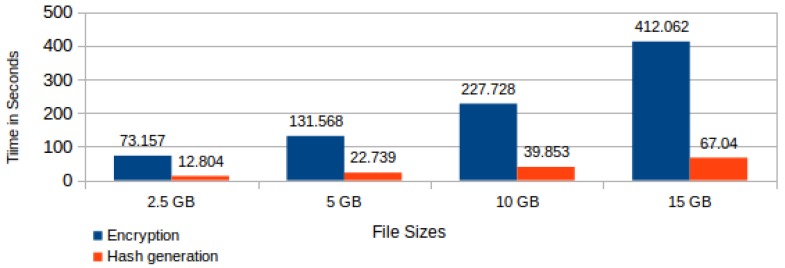
Average time for file encryption and hash generation by file size.

Each of these tasks has its execution time varying according to the processed file size or the foreseen time period for its storage in the CSS. We present hereafter average times taken from a number of 20 controlled repetitions of the experiment or test. Figure 10 shows both the encryption and the hash generation average time by file sizes.

**Figure 11:**
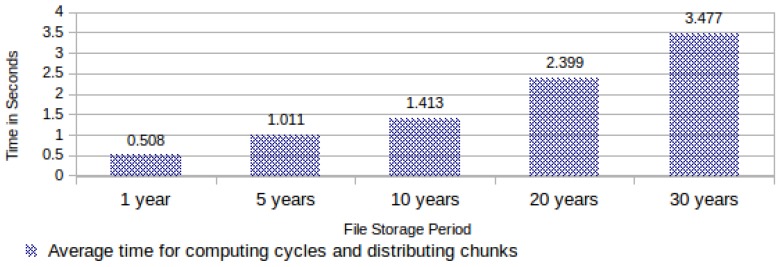
Required average time for computing cycles and distributing chunks.

As explained before, the task “computing cycles and distributing chunks” is responsible for selecting 16 chunk address codes for each data block required for filling the computing cycles. Hence, its execution time varies exclusively according to the file storage period. Figure 11 shows the required average time for computing cycles and distributing chunks.

**Figure 12:**
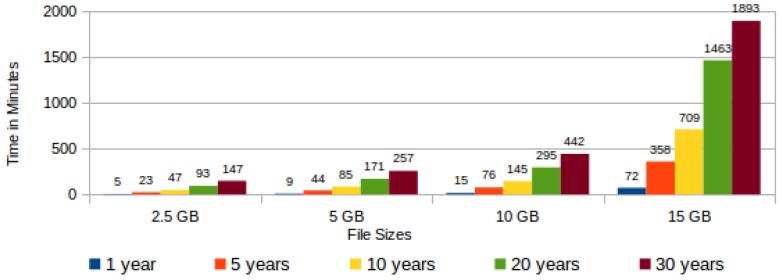
Average time for data block hashing.

The task “hashing data blocks” comprises actions for randomly reading the chunks in the encrypted file, assembling data blocks by the concatenation of chunks and, finally, the hash generation from each assembled data block. In this task, the execution time varies according to both the file size and the expected cloud storage time. The larger the file, the larger will be the size of each data block. Additionally, the longer the storage period, the greater the quantity of data blocks to be generated. Figure 12 shows a graph with the time variation for generating data block hashes according to the file size and the chosen storage period.

Observing Figure 12, it is possible to verify that the time for generating all hashes from a 15 GB file presents a disproportionate growth when compared with the other file sizes, independent of the cloud storage period. From this observation, it is possible to infer that for files with sizes of the order of 15 GB or greater it is necessary to optimize the proposed protocol.

**Figure 13:**
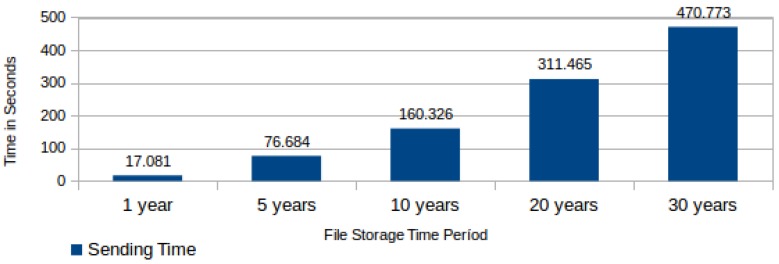
Required average time for sending an information table to the integrity check service (ICS).

Another important component in the file submission process total execution time is the time required to send the information table to the ICS. This time varies according to the quantity of generated data blocks, which in turn varies according to the storage period for the file in the CSS. The measured time in this task comprises the connection with web service in the ICS, the sending of the information table, its storage in the ICS database and the receiving of a successful storage confirmation from the ICS. Figure 13 shows the required average time for sending an information table to the ICS according to the CSS storage period.

### 6.3. Network Bandwidth Consumption

We show results from experiments realized to determine the effective consumption of network bandwidth by the file monitoring process execution. By design, the TOPMCloud implies a variation in network resource consumption according to the trust level assigned to the CSS.

**Figure 14:**
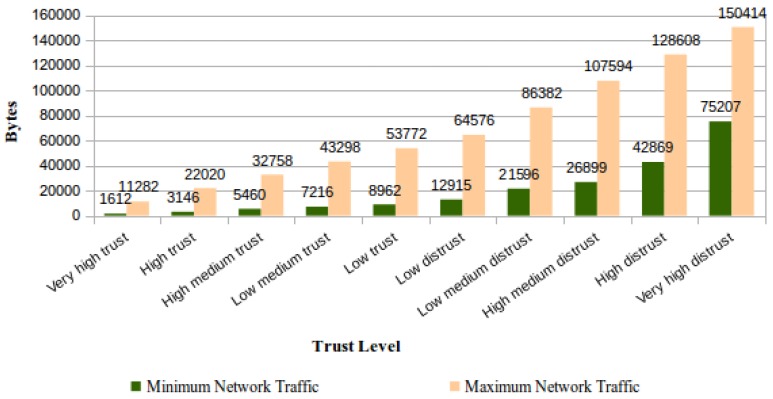
Average daily network bandwidth consumption by stored file.

For evaluating this feature, each trust level foreseen in TOPMCloud was successively assigned to a CSS, and for each assigned level, the ICS performed daily file verifications on the said CSS. The measurements regarding the traffic due to challenges sent by the ICS and corresponding answers were based on traffic collection with the Wireshark tool. Using a controlled network environment and with filters applied on Wireshark, it was possible to exclusively capture packets generated either by the concerned ICS and the CSS applications. Figure 14 shows the average daily network bandwidth consumption by stored file according to the trust level assigned to the CSS.

The rate of network traffic per stored files attains its maximum value when the number of stored files in the CSS is equal to one since the ICS is required to use network bandwidth just for this file. In this case, at least one of these file data blocks will be verified per day, independent of the trust level assigned to the CSS. If this trust level is higher and there is a set of files to be verified, the network traffic will serve to monitor percentages of these files’ contents. The network traffic per stored file always attains its minimum when an integer value is attributed to the percentage computation defined at Table 1, column “Files verified by day”, for the number of files stored in the CSS, according to its trust level.

### 6.4. File Integrity Checking by the ICS

As mentioned before, this operation of the ICS was monitored for three months, in a setup involving one ICS and three CSS. During that period, either the ICS or the CSS stored their logs in text files, with each log record containing the execution time spent with the actions related to the file integrity-checking process.

**Figure 15:**
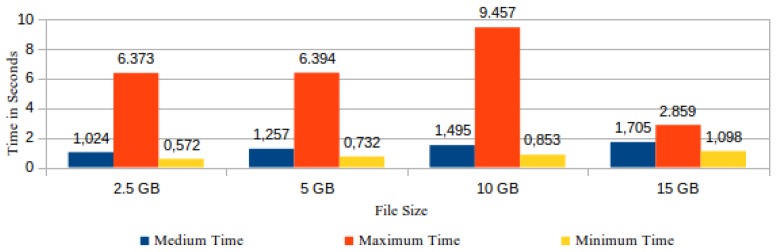
Time spent to conclude the processing of a challenge by file size.

As the file integrity checking is executed by means of challenges sent by the ICS to the CSS, the response time is directly proportional to the verified file size. Thus, the information about challenge results obtained from the ICS logs was grouped and classified according to verified file size. The registered values in the ICS logs comprise the executed actions since the moment of storing the challenge in the “requests” table up to the receiving of the respective answer sent by the CSS. In our experiment, the ICS logs registered 1340 records of successful checking results. Figure 15 shows the medium, maximum and minimum time spent to process a challenge by the verified file size.

**Figure 16:**
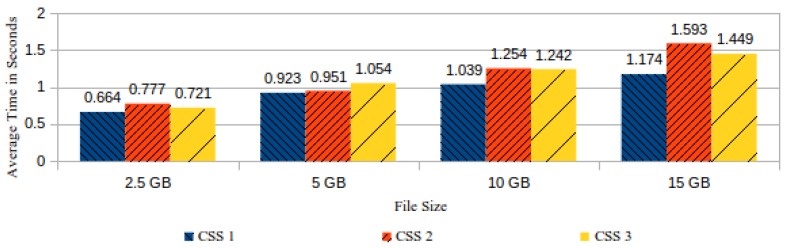
Average time spent by the cloud storage service (CSS) to answer ICS challenges, by CSS and file size.

The most time-consuming actions in the processing of a challenge are realized in the CSS and comprise the reassembly of the data block from the stored file chunks, followed by the task of data block hashing. From the three CSS used in our experiment, the number of records collected in their logs was respectively 360, 470 and 510. These records registered the time spent by each CSS in the execution of the aforementioned actions. In spite of the same number of files having been stored in all three CSS, the number of processed challenges for each CSS varied, because each CSS was randomly turned down for a random time interval. Figure 16 shows the comparison between the average time spent by each CSS answering the requested challenges coming from the ICS.

### 6.5. Simulation of File Storage Faults

The simulation of integrity faults in files stored in the CSS was performed by means of the controlled modification of bytes within some stored files. This simulation purpose was to determine how much time is required by the ICS to identify a fault. For this simulation, ten out of the fifteen “5 GB checked files” were chosen, and then, these files were randomly distributed in two groups each one possessing five files, which had in common the same number of modifications in their bytes.

In File Group 1, 5368 sequential bytes (0,0001%) were changed in each file, and the position for this modification inside each file was randomly chosen. In Group 2, in each file, a number of 5,368,709 sequential bytes (1%) was changed at the end of the file.

**Figure 17:**
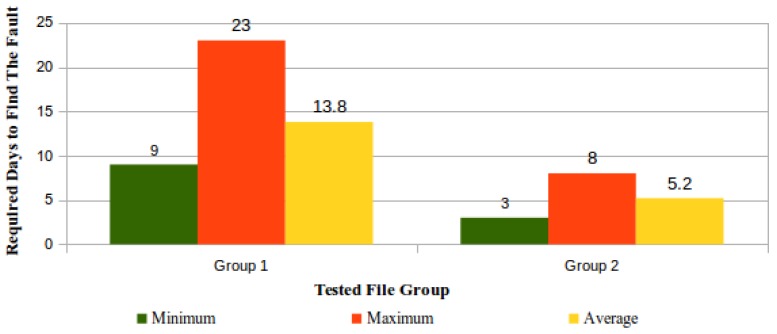
Detection by the ICS of integrity faults in files stored in a CSS.

Figure 17 shows the results regarding the perception of these faults by the ICS.

## 7. Discussion

The related research works described in Section 3 present viable alternatives that respond to the problem of verifying the integrity of files stored in the cloud, but none of them presents a unique and complete solution that allows: permanent and active monitoring, execution by an independent third party, file integrity verification without access to file content, low and predictable computational cost and balanced computational resources’ consumption according to the measured CSS QoS. The main differences between the protocol proposed in this work and those of the cited related works is further discussed as follows.

In [25,26], small changes in files cannot be detected because only a few specific bits in each file are tested, while in our TopMCloud, all bits are tested. In this case, our solution benefits from the integrity check proposed and tested according to the presented results.

In [27], the proposed solution requires a trusted third party to save pieces of information about the files. The trusted third party is necessary because it is not possible to restore the original file content without the saved information. Oppositely, in TopMCloud, the third party can be untrusted because it verifies the file integrity without having direct access to the encrypted file or any information related to its original content.

In [28,29], it is necessary to retrieve the whole ciphered file to verify its integrity, while in TopMCloud, the monitoring is constantly performed without retrieving from the CSS any bit of the stored file. In this case, our solution reduces bandwidth consumption when network traffic is to be considered and still guarantees the integrity of the files when their storage time has to be taken into account, according to the client needs.

In [30,31], the monitoring solutions are based on asymmetric homomorphic algorithms, a type of cryptography scheme that consumes large amounts of computational resources. In TopMCloud, hashes perform faster and consume fewer resources. Thus our solution benefits from speed in processing large files and being able to maintain integrity and confidentiality.

Another interesting consideration is that none of the related works were designed to monitor large files with 5, 10, 15 GB or more than that. Consequently, among the reviewed publications, there were none presenting test results analogous to those coming from the TopMCloud validation process. For this reason, it was not possible to perform a qualitative analysis of the results obtained with the tests applied in TopMCloud in comparison to the mentioned related works.

## 8. Conclusion and Future Work

In this paper, a distributed computational architecture was proposed aimed at monitoring the integrity of stored files in a Cloud Storage Service (CSS) without compromising the confidentiality of these files’ contents. The proposed architecture and its supporting protocol leverage the notion of trust so that a CSS consumer uses a third-party file-checking service, the Integrity Check Service (ICS), to continuously challenge the CSS provider regarding the integrity of stored files and, based on these verifications, to present a level of trust attributed to this CSS provider.

Based on the behavior of each CSS, the file-checking frequency adapts dynamically, either increasing if stored file integrity failures are observed or decreasing if a low failure rate is observed. Consequently, the verification effort is oriented toward being more intensive when it is effectively needed, thus optimizing computational and network resource consumption regarding the proposed protocol’s execution.

The proposed protocol was also designed to address requirements such as low bandwidth consumption, capacity to quickly identify misbehaving storage services, strong resistance against fraud, reduced CSS overhead, confidentiality of stored file contents and capacity to provide predictability and maximum resource savings for the ICS.

Another view of our proposal is that it was designed to provide an efficient control over the integrity of files stored in a CSS without overloading the service providers that present appropriate behavior, but quickly acting if this behavior becomes problematic, which requires our architecture to identify faults and provide early alerts about corrupted files to their owners. These are the main reasons for choosing in our design to perform file integrity monitoring by means of the hashing of stored file parts, without that ICS needing to directly access file contents and avoiding the CSS from processing the complete file on each checking.

The design choice of hashing and verifying data blocks, which are assembled from randomly-chosen file chunks, was demonstrated in our experimental validation as an effective method to detect the simulated file integrity faults that were injected in the CSS under test. Even small modifications in very large files took an average of 14 days to be identified.

Furthermore, based on the test results in our experimental setup, it was possible to verify that the time taken to generate a File Information Table, as well as the size of this table, can be considered adequate, being proportional to the file size. The network bandwidth consumption for the monitoring was very low regardless of the trust level assigned to a CSS.

Another feature of the proposed architecture is that if necessary, it can be used to avoid the CSS consumer needing to store copies of files that are stored in the cloud since the consumer is ensured the capacity to retrieve the files from multiple CSS providers, which constitutes a redundant cloud storage configuration. In this case, the redundancy level must be appropriately chosen by the storage client according to the file information criticality and using the measurements made by the ICS regarding the trust in each CSS used. The CSS classifications in trust levels, according to their availability and their stored file integrity history, besides being applied to relieve computational load for a well-behaving CSS, also allow the clients to use this information to critically select the most suitable CSS to be contracted, according to the information criticality level associated with the files that will be stored in the cloud.

The proposed architecture proved to be quite robust during the tests, satisfactorily responding to the fault simulations. Interestingly enough, the developed prototype also resisted failures not foreseen in the protocol very well, such as unplanned server shutdown (due to electricity outages), not requiring any human intervention for the functionalities to return after restarting the system.

As future work, it is intended to add a functionality that allows the sharing of the measured CSS trust level between different ICS. This functionality would allow, for example, that a fault identified in a CSS by an ICS would alert others ICS so that they can pro-actively react, prioritizing the checking of files stored in that CSS.

Aiming to obtain better performance with files larger than 30 GB, it is intended to test the modifications of our protocol parameters, such as increasing the number of chunks per file and/or the number of chunks per data blocks. In this same sense, it is also intended to improve the architecture implementation in order to configure different processing parallelism schemes.

## Figures and Tables

**Table 1:** Classification of the trust levels for updating purposes.

Observed Trust Level	Value Range	Percentage of Files to be Checked by Day	Percentage of File Contents to be checked by Day	Data Blocks Verified by Day
Very high trust	]0.9, 1[	15%	∼ 0.4%	1
High trust	]0.75, 0.9]	16%	∼ 0.8%	2
Medium-High trust	]0.5, 0.75]	17%	∼ 1.2%	3
Low-medium trust	]0.25, 0.5]	18%	∼ 1.6%	4
Low trust	]0, 0.25]	19%	∼ 2.0%	5
Low distrust	]−0.25, 0]	20%	∼ 2.4%	6
Low medium distrust	]−0.5,−0.25]	25%	∼ 3.2%	8
Medium-high distrust	]−0.75, −0.5]	30%	∼ 4.0%	10
High distrust	]−0.9, −0.75]	35%	∼ 4.8%	12
Very high distrust	]−1, −0.9]	50%	∼ 5.6%	14
